# Insulin Independence in Newly Diagnosed Type 1 Diabetes Patient following Fenofibrate Treatment

**DOI:** 10.1155/2020/6865190

**Published:** 2020-05-14

**Authors:** Karsten Buschard, Laurits J. Holm, Ulla Feldt-Rasmussen

**Affiliations:** ^1^The Bartholin Institute, Department of Pathology, Rigshospitalet, Copenhagen University Hospital, Copenhagen, Denmark; ^2^Department of Medical Endocrinology and Metabolism, Rigshospitalet, Copenhagen University Hospital, Copenhagen, Denmark

## Abstract

A 19-year-old girl was diagnosed with type 1 diabetes and showing polydipsia and polyuria. She was double autoantibody-positive and had a diabetes-prone tissue type. She was immediately started on insulin. Fenofibrate treatment (160 mg daily) was initiated seven days after diagnosis. The need for insulin quickly declined, and she took her last dose of insulin 19 days after the first dose of fenofibrate, having regained endogenous control of blood glucose concentrations. She has now been insulin independent for one year and 9 months. Unstimulated C-peptide has increased by 51% (317 to 479 pmol/l), and IA-2 autoantibody level has decreased by 65% (49 to 17 × 10^3^ arbitrary units). Fenofibrate is a widely used drug for reducing triglyceride and cholesterol levels. Fenofibrate reverses and prevents autoimmune diabetes in nonobese diabetic (NOD) mice by increasing the amount of the sphingolipid sulfatide in islets. Sphingolipid metabolism is otherwise abnormal in the islets at diagnosis of type 1 diabetes. In conclusion, we describe a 19-year-old patient with classical newly diagnosed type 1 diabetes, which following fenofibrate treatment has been without insulin for 21 months.

## 1. Introduction

Type 1 diabetes incidence is increasing worldwide; however, insulin remains the only treatment option [1]. Most clinical trials have focused on modulating the immune response, but have shown limited clinical efficiency, highlighting the need for new prevention and treatment strategies [2]. Various experimental studies have shown that sphingolipids are key regulators of beta-cell biology and inflammation [3–5]. We have recently published abnormal sphingolipid metabolism in islets of newly diagnosed patients with type 1 diabetes [6] with a 77% reduction of the sphingolipid sulfatide in islets and reduced expression of several enzymes involved in sulfatide biosynthesis [6]. The loss of sulfatide may be crucial as sulfatide is known to play various roles in beta cells, regulating insulin folding and secretion, as well as being an immune modulator [7–9]. Furthermore, we identified polymorphisms within the promoter region of eight genes involved in sphingolipid metabolism, all of which are linked to the genetic predisposition of type 1 diabetes (OR up to 1.47) [6]. Finally, we showed that fenofibrate increased the amount of islet sulfatide and completely prevented diabetes in NOD mice. Also, we reversed diabetes in 46% of otherwise diabetic NOD mice treated with fenofibrate after disease onset. Fenofibrate treatment also improved glucose homeostasis in NOD mice [10].

Fenofibrate, belonging to the family of fibrates, was first synthesised in 1974 and has been used for decades to lower triglyceride and increase HDL-cholesterol concentrations [11]. Orally administered fenofibrate is metabolised to fenofibric acid, which is a peroxisome proliferator-activated receptor alpha (PPAR-*α*) agonist. Fenofibrate has a good clinical safety record and has been shown to reduce the development of diabetic retinopathy and other microvascular endpoints in patients with type 2 diabetes [12].

## 2. Case Presentation

A lean 19-year-old Caucasian girl was diagnosed with type 1 diabetes after four weeks of polydipsia and polyuria, blood glucose up to 30.7 mmol/l, glycated haemoglobin (HbA1c) of 124 mmol/mol (13.5%) (reference interval <48 mmol/mol (6.5%)), and minor diabetic ketoacidosis. She was positive for glutamic acid decarboxylase (GAD) autoantibodies >250 × 10^3^ kIU/l (reference interval <5 kIU/l) and insulinoma-associated antigen-2A (IA-2) 49 × 10^3^ arbitrary units/l (reference interval 12 < ×10^3^ arbitrary units/l). Her tissue type is HLA-A^*∗*^02; B^*∗*^08, ^*∗*^15; C^*∗*^01, ^*∗*^07; DRB1^*∗*^03, ^*∗*^04; DQB1^*∗*^02, ^*∗*^03; DPB1^*∗*^04 : 01. There are no cases of type 1 diabetes among the first- or second-degree family, but the mother has autoimmune thyroiditis. She was immediately started on both long- and short-acting insulin related to meals up to 30 units per day. Fenofibrate treatment (160 mg daily) was initiated seven days after the diagnosis. The father of the patient contacted us following the publication of the paper describing the beneficial effects of fenofibrate on NOD mice [6]. The patient obtained the fenofibrate medication herself after consultation with medical doctors. Treatment of type 1 diabetes patients with fenofibrate is allowed according to the Danish laws of free prescription right for medical doctors (Danish Medical Act Paragraph 6). The patient has given oral and written consent to publish her case, and she has reviewed and approved the manuscript.

## 3. Results

**Figure 1:**
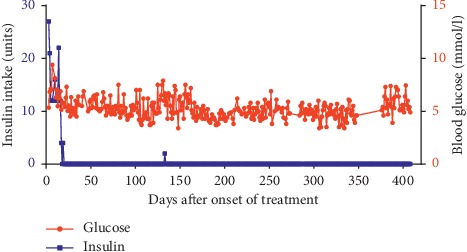
Fenofibrate treatment eliminates the demand for insulin therapy in a newly diagnosed type 1 diabetes patient. A 19-year-old girl diagnosed with type 1 diabetes began taking fenofibrate 160 mg/day seven days after diagnosis. Two units of insulin were taken at day 133 as a precaution when the patient was admitted to a hospital in Sri Lanka with a high fever during her vacation. Data are missing from day 349 to 375 because the patient's computer was stolen. A few other days are missing data. The patient did not take insulin in this period. Graph shows her random mean blood glucose values and insulin intake.

The patient's insulin needs quickly declined, and she took her last insulin 19 days after the first dose of fenofibrate. Blood glucose concentrations were initially between 5 and 10 mmol/l. These tended to be reduced during the next months and has stabilised with a mean concentration of 5 mmol/l, without any insulin treatment (Figure 1). Her HbA1c value decreased to 39 mmol/mol (5.7%) 361 days after diagnosis. Her fasting C-peptide 25 days after the onset of fenofibrate treatment was 317 pmol/l (424 pmol/l glucagon-stimulated value), and this normalised to 479 pmol/l (603 pmol/l glucagon-stimulated value) after 361 days of treatment (reference range 379–1630 pmol/l).

The level of GAD antibodies remained unchanged (>250 × 10^3^ kIU/l), but IA-2 declined to 17 × 10^3^ arbitrary units. Liver and pancreas enzymes were within the normal range. She is feeling completely healthy and only checks her blood glucose concentrations daily as a precaution. The patient took two units of insulin at day 133 after the onset of treatment when she while backpacking in Sri Lanka became sick with a high fever. She took insulin as instructed by doctors as a precaution since type 1 diabetes individuals with fever have a higher demand for insulin. It has now been 21 months since she took her last regular injection of insulin at day 19 after initiation of fenofibrate treatment. She stopped measuring her blood glucose daily after 410 days. However, she still measures blood glucose regularly and is instructed to take insulin if needed. She is examined every third month at Rigshospitalet.

## 4. Discussion

Our patient was diagnosed with classical symptoms of type 1 diabetes; she had a diabetes-prone tissue type, displayed high blood glucose concentrations, and was double autoantibody-positive. She injected her last insulin dose after 19 days of fenofibrate treatment. 1 year and 9 months later, she lives a normal life with normal blood glucose concentrations and fasting and stimulated C-peptide values within the normal range. Spontaneous complete remission cannot be ruled out, but it is rarely observed [13]. Her rise in fasting C-peptide levels of 51% is higher than previously reported [13]. For how long time she will be able to cope without insulin is unknown. To our knowledge, this is the first case of a newly diagnosed individual with type 1 diabetes that has been treated with fenofibrate.
